# Some Diseases of Goats*A Paper read before the British Goat Society

**Published:** 1882-04

**Authors:** A. J. Sewell


### ART. X—SOME DISEASES OF GOATS.*

BY A. J. SEWELL, V.S.

I was of opinion, before I had the pleasure of being veterinary surgeon to this Society, that goats were seldom ill, consequently required very little medicine. I have now come to the conclusion that that was an erroneous opinion, for since being connected with

*A Paper read before the British Goat Society.

the Society I have taken great interest in the diseases affecting this animal, and I find they are very numerous. There is one disease in particular, several cases of which have recently come under my notice. The principal symptoms are continued diarrhœa, heavy breathing, and great emaciation, resulting in many cases in death from exhaustion. I propose to call your attention to this complaint further on. Until our secretary, Mr. Holmes-Pegler, brought out his valuable little handbook on goats, no other book, as far as I know, had ever appeared on the subject, and till very recently the goat was less known about than any other domesticated animal. There is no record of diseases and ailments of goats having been investigated; but perhaps this is from their complaints resembling those affecting sheep; in fact, there are very few diseases affecting the sheep which do not also affect the goat, and *vice versa.* Such diseases as contagious pleuro-pneumonia, cattle plague, foot-and-mouth disease, etc., goats are liable to become attacked with any of these affections as well as cattle, but, of the three, the goat is much less subject to them. This is probably the result of its hardiness; for no animal is more hardy than the goat, when living a natural wild life. Being now more artificially kept in stables and houses erected for the purpose, which, from being over-crowded, want of proper ventilation, and other bad sanitary arrangements, diseases of different kinds are becoming common. The disease I first mentioned, and which has been so fatal amongst the goats of several members, is the one I propose to draw your attention to first.

I do not consider the complaint contagious, though several goats of a flock are often affected at the same time. I think it is the result of some particular grass or condition of the grass on which they are fed. I have not noticed it more prevalent in wet weather than in dry, neither do kids seem to be more susceptible to it than adult goats, as in ordinary diarrhœa; in fact, most of the cases that have came under my notice have been in goats two or three years old. The following are some of the symptoms of the disease:

*Scouring or Diarrhœa.*—This is generally the first symptom noticed, but not always. In some instances it does not come on until the case is somewhat advanced. Then there is loss of appetite, which at first is only slight; but it lessens every day until, in bad cases, the goat refuses food entirely. There is great emaciation, the patient going almost to a skeleton, and looking, if I may so say, like a bag of bones. The breathing is often heavy and laborious, and sometimes offensive, making one think that the lungs were affected. Such, however, is not the case, as I have proved by many *post-mortem* examinations. A cough, as well as a discharge from the nose, is also at times present, but not usually, and only in one or two cases have I observed these two symptoms. The pulse is quick and weak, the conjunctional membranes, or, in plainer language, the internal surface of the eyelids, is pale and anæmic; the gums and inner surface of the lips are in a similar condition; and the tongue is white and furred. The animal stands with arched back, moving only when compelled. The diarrhœa in fatal cases is generally continous throughout the disease.

I will now read extracts from some letters from which I have received from members of the Society respecting the disease in question. One gentlemen, writing last month, says: "I am sorry to say I begin to doubt whether goats will thrive in our meadows, for the grass appears too rich and strong for them. Since writing you last, I have had the misfortune to lose my he-goat, in spite of every care and attention. This makes the second that has died within the last twelve months. I noticed for some weeks that he was gradually getting thin and wasting away. Diarrhœa set in and no treatment would stop it. His appetite was very bad Corn and meal of different kinds, and a variety of green food, were offered him, but he did not seem to care for any, and gradually pined away."

Another member writes: "I had four nannies, two or three kids, and a he-goat. This morning I found one of my nannies dead, and a day or two ago I found a kid dead. They had been suffering from diarrhœa." This writer does not mention any other symptoms, but I feel confident that it was the same disease. The gentleman then goes on to say that his "goats have a small paddock to run in during the day, and at night time they are brought into a house, where they receive hay and some corn, a mixture of maize, oats, and barley." This, you will all admit, is very good treatment for goats. One would have thought the corn—especially the maize and barley—would have counteracted any tendency to diarrhœa which the grass might have induced, but such was not the case. The writer continues: "I lost two other goats in a similar way last year, and I cannot help thinking that goats will not do on our rich grass, but require mountainous places, where they can pick their own food". You see, gentlemen, that this writer is of a similar opinion to the previous one. He lost, altogether, from this complaint, five goats—one nanny, one he-goat, and three kids—and, I am sorry to say, was so disheartened by his misfortunes, and well he might be, that he thought of giving up goat keeping entirely. Our secretary, I am also sorry to say, has been equally unfortunate, as he has lost several valuable animals from the same complaint.

*Treatment.*—This is the most important part of all, for the disease is easily detected. Up to the present this has not been so satisfactory as one would have wished, for the large number of four out of every six cases generally prove fatal. Some goats succumb to the disease after a week's illness, others after a month's lingering sickness, and some cases last even longer than this. Having first warmly housed the patient, which is very necessary, a mild dose of purgative medicine should be given. I say a mild dose because the bowels, being in a relaxed condition, are more easily operated upon than if constipation was present. This medicine is given with the idea of removing any irritating matter that may be present in the intestines. Linseed oil or Epsom salts—it does not matter which one is given—will do. The dose of the oil for an adult goat of moderate size is from one and a half to two ounces; the dose of Epsom salts is about an ounce. This is best given in a little warm oatmeal gruel, the oatmeal assisting the action of the medicine. After the purgative medicine has had time to operate, astringents must be given. The following is what I have used with the best success, namely, powdered catechu, chalk, and gum, of each half a drachm; powdered ginger, one scruple; and powdered opium, six grains. This is one dose, and should be given in a little wheaten-flour gruel, and be repeated two or three times a day, according to the severity of the diarrhœa. As the diarrhœa lessens, the doses should be gradually decreased. It is a bad plan to discontinue the medicine directly the diarrhœa appears to have stopped, for a recurrence of it is often the result. The dose for kids (of the purgative medicine as well as the astringent) is from one-third to a half of that recommended for goats. When the diarrhœa is obstinate, I have given bark with very good success. I have also given this medicine if the appetite is very bad after the diarrhœa-mixture has been discontinued, as it acts as an as_ tringent as well as a good tonic. The dose is about thirty grains of the powdered yellow cinchona bark. This medicine is also best given in gruel. I have heard that port wine and cayenne pepper is a capital mixture for diarrhœa. I have never tried it, but should think it would act very well in mild cases. I saw a prescription for this complaint in *The Live Stock Journal* a few weeks ago. It was "linseed oil two tablespoonfuls, and turpentine one tablespoonful." It sounds rather warm. I should be afraid to give it myself, as I think it would probably increase the irritation of the bowels. Nitrate of silver I have used with very good results in bad cases. When the motions are accompanied with blood, constituting dysentery the dose is a grain and a half to two grains twice a day. It is best given as a pill or bolus made with bread crumbs. I know it is not usual to give a bolus to ruminating animals, but I have often given a small pill in cases like this with good effect.

*Food.*—As to the food, all green food should be withheld, but good meadow hay may be given, and a few oats mixed with barley meal may also be offered. When the patient will not eat, it should be drenched with good gruel made of wheaten flour. As to the quantity that may be given, half a pint three or four times a day is sufficient, for it is a bad plan to overload the stomach. If expense is not objected to, a wineglassful of port wine with each dose of gruel may be added with advantage.

*Foot-Rot.*—There is another troublesome disease that occasionally affects our goats, but it is not a fatal one—at any rate, seldom so. The complaint I mean is foot-rot. I saw a very bad case a few weeks ago. The subject was a good specimen of the Irish breed. The goat, which was a billy, when I first saw him, was unable to put the fore-feet to the ground, but was going about on his knees, which were becoming sore in consequence. The feet had been bad some weeks, and I am sorry to say little attention had been paid him, but I am glad to say that he is now all right. Foot-rot was once, and not very long ago, considered contagious, but it is now considered a non-contagious disease, and that its sole cause is the result of goats (sheep are more liable to it than goats) being kept on low wet ground. In this disease the horn becomes soft, cracks, and the crust turns inwards on the sole. Dirt and grit get between the cracks, and inflammation of the sensitive laminæ, or quick, is the result, causing swelling and tenderness of the foot or feet, and lameness, which at first is only slight, but becomes, if the case is neglected, greatly increased. In these cases the cracks emit an offensive discharge, the coronet, or that part just above the hoof, becomes inflamed, swollen and sore, and the consequence is, an imperfect horn is produced. All four feet are liable to be affected, but the fore feet are more predisposed than the hind feet, probably from their having to bear more weight. Sometimes only one fore foot is bad, and at times only one digit of a foot.

*Treatment.*—When a goat is noticed to be lame, it should be caught and the foot examined. If affected with this disease, it should be well washed with warm water, to which a little Condy's Fluid should be added. All dirt must be removed, as well as all loose, broken, and diseased horn. This is best done with a sharp knife; a small one used by farriers, and called a "searcher," answers the purpose very well. Care must be taken when paring the horn not to draw blood. Hot linseed poultices should be applied to the affected feet for a couple of days. This helps to remove the soreness and imflammation. Then one of the following dressings should be smeared over the diseased parts daily: Sulphate of copper, 1 oz.; lard, 3 oz.; or carbolic acid, 1 dram; olive oil, 1 oz.

Sometimes ulcers and fungoid growths appear in the interdigital spaces. These may be carefully touched with the following strong tincture: Nitric acid and compound tincture of myrrh, of each 2 drams; and spirits of wine, 1 oz. Of course before commencing to treat a case of foot-rot, the patient should be removed to a dry place. This precaution is absolutely necesary. After the worst symptoms of the disease have passed, the following ointment, well rubbed into the hoof daily, stimulates the growth of horn, and keeps it soft and healthly. The ointment is composed of Stockholm tar, one part; and tallow, two parts. The last ingredient should be melted, and then the tar added, and well mixed by stirring. I hope at some future time to have the pleasure of bringing under your notice other diseases affecting the goat.

